# Impact of IFN-γ Deficiency on the Cardiomyocyte Function in the First Stage of Experimental Chagas Disease

**DOI:** 10.3390/microorganisms10020271

**Published:** 2022-01-25

**Authors:** Danilo Roman-Campos, Policarpo Sales-Junior, Alexandre D. Costa, Diego Santos Souza, Artur Santos-Miranda, Julliane V. Joviano-Santos, Catherine Ropert, Jader S. Cruz

**Affiliations:** 1 Laboratório de Cardiobiologia, Department of Biophysics, Federal University of São Paulo, São Paulo 04021, Brazil; santos.dss@outlook.com (D.S.S.); santosmirandaa.edu@gmail.com (A.S.-M.); jullianejoviano@hotmail.com (J.V.J.-S.); 2 Oswaldo Cruz Foundation, Eusébio 61760, Brazil; policarpoasjunior@yahoo.com.br; 3 Laboratório de Membranas Excitáveis e de Biologia Cardíaca, Department of Biochemistry and Immunology, Federal University of Minas Gerais, Belo Horizonte 31270, Brazil; alexandrefiufv@gmail.com (A.D.C.); ropertcatherine@gmail.com (C.R.)

**Keywords:** Chagas disease, cardiomyocytes, L-type calcium current, interferon gamma

## Abstract

Chagas disease (CD) is caused by the parasitic protozoan *T. cruzi.* The progression of CD in ~30% of patients results in Chagasic Cardiomyopathy (CCM). Currently, it is known that the inflammatory system plays a significant role in the CCM. Interferon-gamma (IFN-γ) is the major cytokine involved in parasitemia control but has also been linked to CCM. The L-type calcium current (I_Ca,L_) is crucial in the excitation/contraction coupling in cardiomyocytes. Thus, we compared I_Ca,L_ and the mechanical properties of cardiomyocytes isolated from infected wild type (WT) and IFN-γ^(−/−)^ mice in the first stage of *T. cruzi* infection. Using the patch clamp technique, we demonstrated that the infection attenuated I_Ca,L_ in isolated cardiomyocytes from the right and left ventricles of WT mice at 15 days post-infection (dpi), which was not observed in the IFN-γ^(^^−/−^^)^ cardiomyocytes. However, I_Ca,L_ was attenuated between 26 and 30 dpi in both experimental groups. Interestingly, the same profile was observed in the context of the mechanical properties of isolated cardiomyocytes from both experimental groups. Simultaneously, we tracked the mortality and MCP-1, TNF-α, IL-12, IL-6, and IL-10 serum levels in the infected groups. Importantly, the IFN-γ^(−/−)^ and WT mice presented similar parasitemia and serum inflammatory markers at 10 dpi, indicating that the modifications in the cardiomyocyte functions observed at 15 dpi were directly associated with IFN-γ^(^^−/−^^)^ deficiency. Thus, we showed that IFN-γ plays a crucial role in the electromechanical remodeling of cardiomyocytes during experimental *T. cruzi* infection in mice.

## 1. Introduction

Chagas disease (CD) is a significant disease in Latin America and other regions of the world caused by the intracellular protozoan parasite *Trypanosoma cruzi* [1,2]. In fact, CD is considered a debilitating disease that accounts for the most significant morbidity and mortality among parasitic diseases [3]. Innate and adaptive immune responses are critical for infection control, and the connection between these responses is mainly orchestrated by cytokines. In this regard, the interferon-γ (IFN-γ) type II subfamily plays a vital role [4]. Furthermore, increased circulating IFN-γ levels correlate with the electromechanical remodeling of both left and right ventricular cardiomyocytes during the acute phase of CD, as well as CD progression in humans [5,6,7]. Thus, we hypothesized that reducing IFN-γ production may ameliorate the cardiomyocyte remodeling observed in the acute phase of experimental CD.

## 2. Materials and Methods

Eight-week-old male C57BL/6 (WT) and IFN-γ^(^^−/−)^ mice were intraperitoneally infected with 50 bloodstream trypomastigote forms of the Colombian strain (DTU—TcI) of *T. cruzi*. A mouse inflammation kit from BD^TM^ (San Jose, CA, USA) was used to detect the serum cytokine and chemokine levels. Prof. Dr. Ricardo T. Gazzinelli generously provided the IFN-γ^(^^−/−)^ mice. All animal experiments were approved by the local ethical committee (number 31/08) and conducted according to the American Association for Laboratory Animal Science guidelines [8]. The WT and IFN-γ^(^^−/−)^ mice were kept under the same conditions during infection at the animal facilities of the René Rachou Center (FIOCRUZ, Brazil) and were maintained in pathogen-free conditions. For euthanasia, the animals were taken to the laboratory for further analysis. A set of mice was used for survival curve, cytokine, and chemokine measurements, and another set of mice was used for the in vitro experiments. Left and Right Ventricular Cardiomyocytes (LVC and RVC) from age-matched mice were enzymatically isolated as previously described in [9]. Only calcium-tolerant, quiescent, rod-shaped myocytes showing clear cross-striations were investigated. Cardiomyocytes isolated from the non-infected and infected mice were studied 12–15 days post-infection (dpi) or 26–30 dpi.

We performed cell contraction analysis using an NTSC camera (MyoCam, Ionoptix, Milton, MA, USA) and pacing cells at a 1-Hz frequency at room temperature, as previously described in [10]. Briefly, the isolated cells were placed in a chamber with a glass coverslip base mounted on the stage of an inverted microscope. During measurement the chamber was perfused with Tyrode’s solution of (in mM) 140 NaCl, 5.4 KCl, 1 MgCl2, 1.8 CaCl2, 10 HEPES, and 10 glucose (pH set at 7.4).

Whole-cell voltage clamp recordings were obtained using an EPC-9.2 patch clamp amplifier (HEKA Electronics, Germany). After gaining access to the whole-cell configuration, the pipette solution was allowed to equilibrate with the cell cytoplasm for 2–3 min. The resistances of the recording electrodes ranged from 1 to 2 MΩ. The current recordings were low-pass filtered (2.9 kHz) and digitized at 10 kHz before being stored on a computer for offline analysis. Cardiomyocytes showing a series resistance larger than 8 MΩ were excluded from further analysis. The electronic compensation of series resistance was set at 40–70%.

For measurements of the L-type Ca^2+^ current (I_Ca,L_), the patch pipettes were filled with (in mM) 120 CsCl, 20 TEACl, 5 NaCl, 10 HEPES, and 5 EGTA with the pH set to 7.2 with CsOH. I_Ca,L_ was measured using Tyrode’s solution as an external solution. The membrane potential was first stepped from a holding potential of −80 mV to −40 mV for 50 ms (to inactivate the voltage-gated Na^+^ channels) and then stepped to different membrane potentials between −40 mV and 50 mV (300-ms duration). All currents were analyzed in terms of peak current (pA) normalized by the cell capacitance (pF). All experiments were conducted at room temperature (~25 °C). All salts used in the manuscript were purchased from Sigma Aldrich (Saint Louis, MO, USA).

Data were expressed as mean ± standard error. The statistical significance of the parametric data between multiple groups was determined by one-way or two-way ANOVA, followed by Bonferroni’s post-test. N and n represent the number of animals and cells, respectively, and are given in each figure legend. We analyzed the mortality curve using the Kaplan–Meier plot. Comparisons were considered statistically significant when *p* < 0.05.

## 3. Results

**Figure 1:**
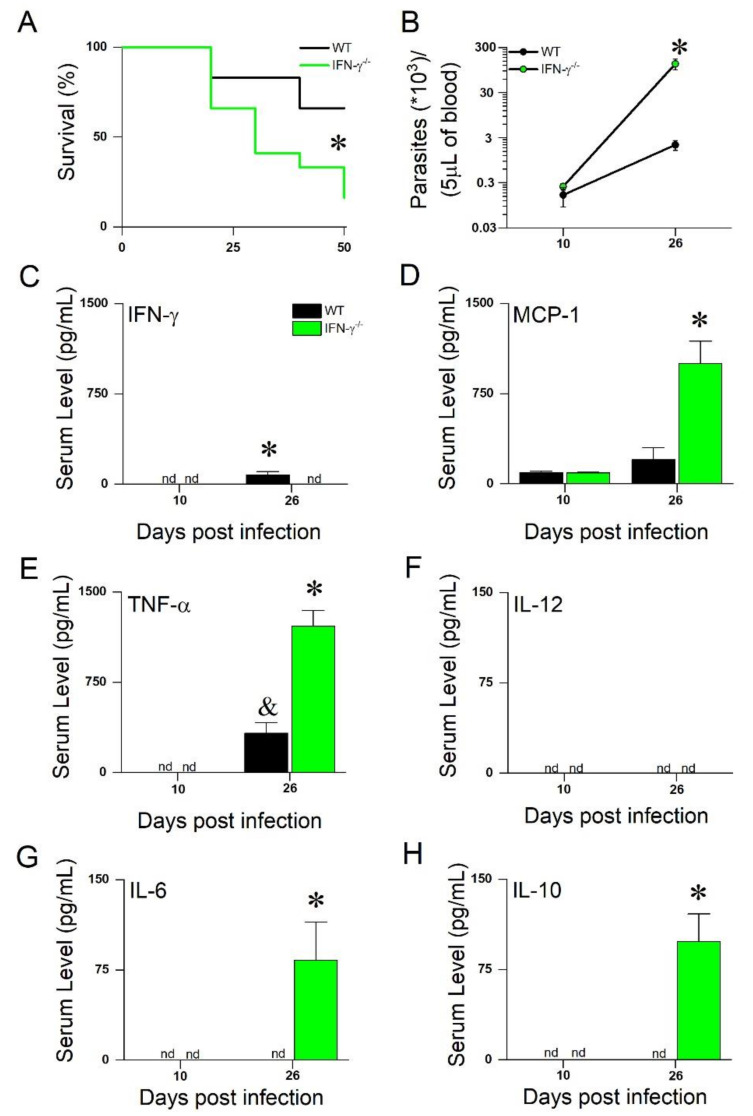
(**A**) Mortality curves were created using 12 animals/group. (**B**) Parasitemia curves were taken from WT (N = 6) and IFN-γ^(^^−/−)^ (N = 8) mice. Quantification by CBA cytometric bead array of IFN-γ (**C**), MCP-1/CCL2 (**D**), TNF-α (**E**), IL-12 (**F**), IL-6 (**G**), and IL-10 (**H**). N = 3–5 animals, *p* < 0.05. * = different from all. & = different from WT at 10 dpi. Mortality curves were analyzed using Kaplan–Meier estimator. Two-way ANOVA used for analysis of parasite and cytokine or chemokine levels, comparing time course infection of WT and IFN-γ^(^^−/−)^ mice. nd = not detected.

The survival of the WT and IFN-γ^(^^−/−)^ mice was monitored for the first 50 days following infection (Figure 1A, 50 dpi), and as expected, the IFN-γ^(^^−/−)^ mice exhibited a higher mortality rate than the WT mice. At 10 dpi, the parasitemia levels in both groups were comparable. However, at 26 dpi, the IFN-γ^(^^−/−)^ mice exhibited almost 100-fold more parasites in the bloodstream (Figure 1B). At 10 dpi, the cytokines and chemokines evaluated in this study were not detected in the WT or IFN-γ^(^^−/−)^ mice, except for MCP-1, which had similar serum levels in both groups (Figure 1C–H). However, at 26 dpi, we observed a different inflammatory profile compared with the IFN-γ^(^^−/−)^ and WT mice, with significant increases in the MCP-1, TNF-α, IL-6, and IL-10 levels (Figure 1C–H). Next, we examined the isolated cardiomyocytes at 15 and 30 dpi to determine whether the onset of cardiomyocytes’ electromechanical remodeling would have preceded the increased level of circulating cytokines observed at 26 dpi (see Figure 1).

**Figure 2:**
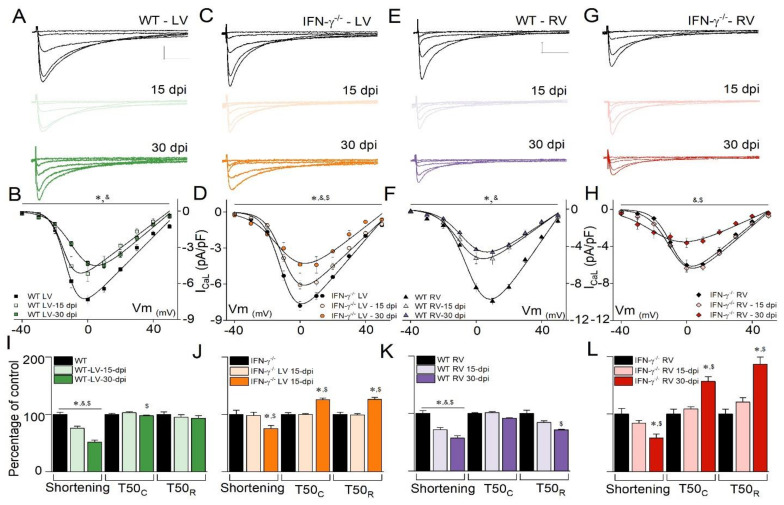
Representative L-type Ca^2+^ current (I_Ca,L_) traces for WT left (**A**) and right (**E**) ventricular cardiomyocytes and IFN-γ^(−/−^^)^ left (**C**) and right (**G**) ventricular cardiomyocytes at 0, 15, and 30 days post-infection (dpi). Scale bar for I_Ca,L_ is equal for all traces. Current density–voltage relationships for tested membrane potentials in WT left (**B**) and right (**F**) ventricular cardiomyocytes and IFN-γ^(−/−^^)^ left (**D**) and right (**H**) ventricular cardiomyocytes. WT-LVCs (N = 4, *n* = 8), WT-LVCs-15-dpi (N = 3, *n* = 7), WT-LVCs-30 dpi (N = 3, *n* = 6), WT-RVCs (N = 3, *n* = 7), WT-RVCs-15 dpi (N = 4, *n* = 10), WT-RVCs-30 dpi (N = 5, *n* = 8), IFN-γ^(−/−^^)^-LVCs (N = 4, *n* = 10), IFN-γ^(−/−^^)^-LVCs-15 dpi (N = 3, *n* = 15), IFN-γ^(−/−^^)^-LVCs-30 dpi (N = 3, *n* = 7), IFN-γ^(−/−^^)^-RVCs (N = 6, *n* = 18), IFN-γ^(−/−^^)^-RVCs-15 dpi (N = 4, *n* = 16), and IFN-γ^(−/−^^)^-RVCs-30 dpi (N = 3, *n* = 6). Cell shortening and time to 50% contraction (T50_C_) and relaxation (T50_R_) normalized as a function of respective control for WT left (**I**) and right (**K**) ventricular cardiomyocytes and IFN-γ^(−/−^^)^ left (**J**) and right (**L**) ventricular cardiomyocytes. WT-LVCs (N = 3, *n* = 29), WT-LVCs-15 dpi (N = 5, *n* = 39), WT-LVCs-30 dpi (N = 6, *n* = 51), WT-RVCs (N = 3, *n* = 27), WT-RVCs-15 dpi (N = 4, *n* = 30), WT-RVCs-30 dpi (N = 5, *n* = 39), IFN-γ^(−/−^^)^-LVCs (N = 6, *n* = 43), IFN-γ^(−/−^^)^-LVCs-15 dpi (N = 4, *n* = 55), IFN-γ^(−/−^^)^-LVCs-30 dpi (N = 4, *n* = 47), IFN-γ^(−/−^^)^-RVCs (N = 4, *n* = 39), IFN-γ^(−/−^^)^-RVCs-15 dpi (N = 4, *n* = 45), and IFN-γ^(−/−^^)^-RVCs-30 dpi (N = 5, *n* = 36). Continuous lines (**B**,**D**,**F**,**H**) are the best fits using the Boltzmann function. Scale is the same for all traces, where x = 500 ms, y = 2.5 pA/pF, and *p* < 0.05. * = comparing non-infected with 15 dpi. & = comparing non-infected with 30 dpi. $ = comparing 15 dpi with 30 dpi. Two-way ANOVA used for I_Ca,L_ and one-way ANOVA for cell shortening experiments. LV = left ventricle. RV = right ventricle.

To study this further, we investigated the time course of the I_Ca,L_ changes for LVC. Figure 2A,B (WT) and Figure 2C,D (IFN-γ^(^^−/−)^) depict the representative traces of I_Ca,L_ and graphs. *T. cruzi* infection at 15 and 30 dpi reduced the I_Ca,L_ peak current density in both experimental groups. However, the analysis of the current vs. voltage relationships for I_Ca, L_ revealed that the diminution of the I_Ca,L_ density in infected IFN-γ^(^^−/−)^ mice (Figure 2D) at 15 dpi, was not as large as that of the infected WT mice (seen in Figure 2B). Normalized conductance curves were analyzed, and WT-LVC-30 dpi showed a right shift in the steady state activation relationship, which was not observed for IFN-γ^(^^−/−)^-LVC-30 dpi (Supplementary Materials Table S1).

Using the same electrophysiological protocol, we assessed I_Ca,L_ in RVC from the WT and IFN-γ^(^^−/−)^ mice, and Figure 2E–H depict the representative recordings and graphs. Evaluation of the I_Ca,L_ current–voltage relationship in WT-RVC indicated a decrease in I_Ca,L_ at 15 dpi (Figure 2F). However, in IFN-γ^(^^−/−)^-RVC, the reduction of I_Ca,L_ was observed only at 30 dpi (Figure 2H). Interestingly, normalized conductance curves for WT-RVC showed a left shift in the voltage dependency for current activation, a feature that was not observed in IFN or IFN-γ^(^^−/−)^-RVC (Supplementary Materials Table S2). Since the time course of I_Ca,L_ remodeling was time- and region-dependent after *T. cruzi* infection, and knowing that I_Ca,L_ plays a crucial role in the excitation contraction coupling in the heart muscles, we decided to investigate cardiomyocyte contractility. As indicated in Figure 2I, WT-LVC cell shortening was significantly attenuated at 15 and 30 dpi in WT-LVC but only at 30 dpi in IFN-γ^(^^−/−)^-LVC (Figure 2J). These results correlate with the observed time course of I_Ca,L_. At 30 dpi, the time to 50% relaxation (T50_R_) was significantly prolonged in IFN-γ^(^^−/−)^-LVC but not in WT-LVC. WT-RVC (Figure 2K) and IFN-γ^(^^−/−)^-RVC (Figure 2L) presented similar results.

## 4. Discussion

In this study, we showed that a global absence of IFN-γ during *T. cruzi* infection (1) induced similar parasitemia and cytokine and chemokine levels (TNF-α, IL-6, IL-10, MCP-1, and IFN-γ) in WT and IFN-γ^(^^−/−)^ infected mice in the early stage of infection and (2) delayed the onset of electromechanical remodeling in the LVC and RVC from the mice.

In acute CD, a widespread immunological reaction is characterized by diffuse lymphadenopathy, hepatomegaly, and splenomegaly. Direct tissue parasitism may result in inflammation of tissues that include the heart. IFN-γ production has been consistently associated with altered cardiomyocyte function in the acute phase of experimental CD [10] and with heart malfunction in the chronic phase of CD in humans [6,7]. To investigate the role of IFN-γ in experimental CD, we inoculated a small number of parasites in both WT and IFN-γ^(^^−/−)^ animals to keep them alive longer. Even in this infection model, parasitemia and mortality were significantly higher in infected mice lacking IFN-γ, reemphasizing the crucial role of IFN-γ in infection control. The main observation of this study was that in the early period just after infection, when the cytokine levels were comparable in the WT and IFN-γ^(^^−/−)^ mice (between 10 and 15 dpi), IFN-γ deficiency attenuated the changes in I_Ca,L,_ and cardiomyocyte contractility in both LVC and RVC. This enabled us to hypothesize that IFN-γ has a stage-dependent impact on cardiomyocyte function, with a predominant role at the onset of infection.

It is already known that IFN-γ production in WT mice infected with *T. cruzi* activates the inducible isoform of nitric oxide synthase (iNOS) and, consequently, elevates nitric oxide (NO^●^) production. It is also known that NO^●^ is fundamental for the macrophage trypanocidal activity [11], particularly when combined with the highly produced superoxide anion to form peroxynitrite [12]. An increase in overall NO^●^ production during *T. cruzi* infection depends on IFN-γ, which partially explains the inability of IFN-γ^(^^−/−)^ mice to control infection. Importantly, in previous studies from our group, we reported that NO^●^ was a significant determinant of I_Ca,L_ remodeling in cardiomyocytes from WT at 30 dpi and that iNOS ablation delayed the onset of cardiomyocyte remodeling [13].

Interestingly, a previous report demonstrated that the activation of canonical PI3Kinase-γ signaling in macrophages is fundamental for controlling parasite levels [14], and the same pathway may also contribute to parasite control in cardiomyocytes. In line with this evidence, we demonstrated that during the acute phase of experimental CD, the PI3Kinase-NO^●^ pathway is activated in cardiomyocytes and is partially responsible for the attenuation of I_Ca,L_ in these cells [15]. Nevertheless, the experimental evidence presented in this study suggests that IFN-γ plays a significant role in the electromechanical remodeling of cardiomyocytes at 15 dpi. Lastly, we propose that selectively targeting IFN-γ in the heart tissue might delay the onset of electromechanical remodeling during *T. cruzi* infection while controlling parasite burn.

### Study Limitations

First, during the in vitro experiments, we did not use markers to differentiate *T. cruzi* infected and non-infected myocytes in the infected mice. Thus, our results indicate the direct *T. cruzi* action in the cardiomyocyte or parasite-induced changes in the heart extracellular environment, which may contribute to the electromechanical remodeling observed in cardiomyocytes.

Second, it is worth noting that the absence of IFN-γ in RVC triggered the presence of additional ion conductance at membrane potentials ranging from −30 to −20 mV. Indeed, we observed a left shift in calcium conductance, although it was not significant compared with the control (Supplementary Materials Table S2). One possible explanation is that the re-expression of T-type calcium channels occurs in this specific cell group. However, additional experiments should be conducted to uncover this possibility.

## Data Availability

The datasets generated during and/or analyzed during the current study are available from the corresponding author on reasonable request.

## Supplementary Materials

The following supporting information can be downloaded at: https://www.mdpi.com/article/10.3390/microorganisms10020271/s1; Table S1: Biophysical parameters of L-type Ca^2+^ current steady-state activation in left ventricular cardiomyocytes (LVCs); Table S2: Biophysical parameters of L-type Ca^2+^ current steady-state activation in right ventricular cardiomyocytes (RVCs).
