# Supplementary material for: Impact of IFN-γ Deficiency on the Cardiomyocyte Function in the First Stage of Experimental Chagas Disease
Source: Microorganisms. 2022 Jan 25;10(2):271. doi: 10.3390/microorganisms10020271 (PMC8874532; doi:10.3390/microorganisms10020271)
Supplement: Supplementary file 1 [file microorganisms-10-00271-s001.zip › microorganisms-1510883-supplementary.pdf]

Supplementary Table S1 – Biophysical parameters of L-type Ca^{2+} current steady-state activation in left ventricular cardiomyocytes (LVCs)

WT- LVCs n=9	WT- LVCs -15 dpi n=8	WT- LVCs -30-dpi n=7	IFN- $\gamma^{-/-}$- LVCs n=11	IFN-$\gamma^{-/-}$- LVCs -15-dpi n=16	IFN-$\gamma^{-/-}$- LVCs -30- dpi n=8
$V_{(half)\ (mV)}$					
-12.51±1.21	-13.75±1.12	-7.45±1.24*	-10.51±0.6	-9.11±0.37	-11.97±2.29
Slope factor					
3.64±0.29	3.69±0.39	5.81±0.32*	4.52±0.27	4.66±0.28	7.56±1.18[&]

* compared to WT-LV and WT-LV-15-dpi

[&] compared to IFN- $\gamma^{-/-}$-LV and IFN-$\gamma^{-/-}$-LV-15-dpi

Supplementary Table S2 – Biophysical parameters of L-type Ca^{2+} current steady-state activation in right ventricular cardiomyocytes (RVCs)

WT- RVCs n=7	WT- RVCs -15 dpi n=10	WT- RVCs -30-dpi n=8	IFN-$\gamma^{-/-}$- RVCs n=18	IFN-$\gamma^{-/-}$- RVCs -15-dpi n=16	IFN-$\gamma^{-/-}$- RVCs -30-dpi n=6
$V_{(half)\ (mV)}$					
-3.39 ± 0.85	-6.84 ± 0.84*	-6.03 ± 0.86	-8.22 ± 0.62	-8.32 ± 0.53	-10.59 ±1.75
Slope factor					
5.71 ± 0.12	5.76 ± 0.51	6.12 ± 0.32	4.38 ± 0.13	5.02±0.24	5.96 ± 0.78[&]

*compared to WT-RV

[&] compared to IFN-$\gamma^{-/-}$-RV
